# COVID-19 and Diabetes: Understanding the Interrelationship and Risks for a Severe Course

**DOI:** 10.3389/fendo.2021.649525

**Published:** 2021-06-17

**Authors:** Cyril P. Landstra, Eelco J. P. de Koning

**Affiliations:** Department of Internal Medicine, Leiden University Medical Center, Leiden, Netherlands

**Keywords:** diabetes, COVID-19, SARS-CoV-2, coronavirus, severity, mortality, comorbidities, treatment

## Abstract

The relationship between COVID-19 and diabetes mellitus is complicated and bidirectional. On the one hand, diabetes mellitus is considered one of the most important risk factors for a severe course of COVID-19. Several factors that are often present in diabetes mellitus are likely to contribute to this risk, such as older age, a proinflammatory and hypercoagulable state, hyperglycemia and underlying comorbidities (hypertension, cardiovascular disease, chronic kidney disease and obesity). On the other hand, a severe COVID-19 infection, and its treatment with steroids, can have a specific negative impact on diabetes itself, leading to worsening of hyperglycemia through increased insulin resistance and reduced β-cell secretory function. Worsening hyperglycemia can, in turn, adversely affect the course of COVID-19. Although more knowledge gradually surfaces as the pandemic progresses, challenges in understanding the interrelationship between COVID-19 and diabetes remain.

## Introduction

Diabetes mellitus is a complex chronic disease characterized by glucose dysregulation caused by an absolute or relative insulin deficiency. It includes various different types, with type 1 diabetes (T1D) and type 2 diabetes (T2D) as the most prevalent subtypes. T1D is characterized by autoimmune destruction of insulin-producing pancreatic β-cells, while T2D results from a combination of β-cell secretory defect and insulin resistance (1). The global burden of diabetes is high, with an overall prevalence of 9.3% and 463 million people suffering from the disease worldwide (2). It is often accompanied by various comorbidities and long-term complications, including obesity, hypertension, vasculopathy, a proinflammatory and hypercoagulable state and cardiovascular disease (CVD) (3–5).

In December 2019, the first cases of atypical pneumonia with Severe Acute Respiratory Syndrome Coronavirus 2 (SARS-CoV-2) were identified. A rapid global spread of the virus led the World Health Organization (WHO) to declare coronavirus disease 2019 (COVID-19) a pandemic on March 11^th^, 2020. Clinical presentation of COVID-19 is diverse and can vary from asymptomatic infection and mild upper respiratory tract symptoms to respiratory failure needing intensive care and death.

This review aims to provide an overview and assessment of the bidirectional relationship between COVID-19 and diabetes. On the one hand, diabetes and its associated comorbidities increase the risk of a more severe course of COVID-19 and increased mortality (6–10). Patients with diabetes are known to have an increased risk of infections, which is partly attributed to hyperglycemia causing immune dysfunction, among other effects (11–15). On the other hand, severe SARS-CoV-2 infection and its associated hyperinflammation contribute to hyperglycemia through an indirect negative effect on insulin target tissues and a potential direct negative effect on pancreatic β-cells (16). The resulting hyperglycemia can, in turn, worsen the prognosis of COVID-19 (17–20).

In this review, we will first discuss the primary risk of COVID-19 infection in patients with diabetes, then the risk of a severe course of COVID-19 with diabetes, followed by the potential additional risk of the most relevant comorbidities and other concomitant factors, and finally the role of glycemic control and the effect of the SARS-CoV-2 virus itself. All studies that were considered relevant for this review were found using PubMed and by cross-referencing.

## Risk of Infection With COVID-19

Patients with diabetes are known to have an increased risk of infections, in particular skin infections, genito-urinary tract infections and (bacterial) respiratory tract infections (14, 21). The hyperglycemic environment present in diabetes favors immune dysfunction through several pathways. The most important underlying mechanisms are a decreased production of interleukins in response to an infection, reduced chemotaxis and phagocytic activity, and immobilization of polymorphonuclear leukocytes. Hyperglycemia, including the resulting glycosuria, also increases the virulence of certain pathogens (11, 22, 23). In addition to an increased risk of infection, patients with diabetes also have a higher rate of infection-related hospitalizations as well as infection-related mortality. These risks are present in both T1D and T2D, but are greater in T1D (15). Importantly, it is well-known that the risk of infections is further increased with poorer glycemic control (12–14).

For SARS-CoV-2, clinical reports from all around the world found diabetes mellitus to be one of the most common comorbidities present in patients with COVID-19. In the beginning of the COVID-19 pandemic, this finding, along with the known increased infection risk for other infections, led to the assumption that patients with diabetes are at increased primary risk of COVID-19 infection. However, most of these reports describe patients in a hospitalized or even intensive care unit (ICU) setting, i.e. patients with a more severe course of the disease.

In the first largest consecutive case series from the United States of America (USA), describing 5,700 patients with COVID-19 admitted to 12 hospitals in the New York area, diabetes mellitus was the third most common comorbidity with 33.8% of patients suffering from the disease, after hypertension (56.6%) and obesity (41.7%) (24). This series, however, included both patients admitted to the general ward as well as to the ICU and does not distinguish between the two. A meta-analysis of six Chinese studies including 1,527 patients hospitalized with COVID-19 clearly demonstrated the difference between prevalence of diabetes in severe versus non-severe cases, describing a prevalence of diabetes of 11.7% in ICU cases, but 4.0% in non-ICU cases (25). In another study from China, prevalence of diabetes in 1,590 patients with COVID-19 was 8.2%, rising to 34.6% in patients with a severe course of the disease (26). In line with this finding, a report of the Chinese Center for Disease Control and Prevention on 44,672 COVID-19 cases which also included non-hospitalized patients, showed a lower prevalence of diabetes (5.3%) (27). Similarly, the US Centers for Disease Control and Prevention reported an overall prevalence of diabetes in patients with COVID-19 of 10.9%, with a prevalence of 6.4% in non-hospitalized COVID-19 patients (28). In a meta-analysis on 33 studies with a total of 16,003 patients, which included 30 studies from China, two from the USA and one from France, the pooled prevalence of diabetes in patients with COVID-19 was 9.8% (29). Finally, in Italy, a single-center study on 146 patients hospitalized with COVID-19 demonstrated a prevalence of diabetes of 8.9% (30). With an estimated prevalence of diabetes mellitus in the general population of 10.5% in the USA (31), 11.2% in China (32), 7.6% in France (33), and 8.3% in Italy (34), respectively, the prevalence of diabetes in patients infected with COVID-19 was not higher than in the general population.

In summary, based on these studies, the primary risk of infection with COVID-19 does not appear to be increased in patients with diabetes mellitus. In previous studies with other pathogens, the increased risk of infection in patients with diabetes also appeared to be present predominantly in bacterial, fungal or yeast infections and not in viral infections, such as COVID-19 (13). However, we do not know whether patients with diabetes behave differently with regard to willingness for SARS-CoV-2 testing and/or social distancing compared to patients without diabetes, which could affect incidence rates. Therefore, it is difficult to determine and compare the primary risk of infection. Since most studies involve patients with T2D and/or authors had not specified the type of diabetes, any possible difference between T1D and T2D in primary risk of COVID-19 infection is not yet known.

## Risk of a Severe Course of COVID-19

During previous pandemics, numerous studies have shown that patients with diabetes are a key vulnerable group for a severe course of infections. During the 2009 H1N1 influenza pandemic, hospitalization of individuals with diabetes was up to six times higher as compared to individuals without diabetes (35, 36), risk of admission to the intensive care unit was four times higher (37), and risk of death two times higher (38). In the first SARS-CoV outbreak in 2002, diabetes was determined an independent risk factor for complications and death, with an odds ratio (OR) of 3.0 for death in patients with diabetes (39, 40). During the Middle-East respiratory syndrome coronavirus (MERS-CoV) outbreak, the prevalence of diabetes was 51% in patients who had MERS, and the OR for severe or critical disease ranged from 7.2 to 15.7 in patients with diabetes as compared to the overall population, with a mortality rate of 35% (41).

In the present SARS-CoV-2 pandemic, several studies and meta-analyses have investigated the impact of diabetes on severity of COVID-19. A severe course of COVID-19 is a broad concept, ranging from the need versus no need for hospitalization, ICU versus non-ICU admission, requirement versus no requirement of mechanical ventilation, fatal versus non-fatal disease and any composite outcome combining them. Although chosen endpoints vary per study, there was consensus in currently available literature that in order to be regarded as having a severe course of COVID-19, patients at least have to be hospitalized as a result of the disease. In the initial reports from China, the prevalence of diabetes was consistently higher among patients with severe versus non-severe disease (8, 42–44). As the pandemic progressed, larger studies from countries around the world provided more robust information.

In China, a nationwide study reported a higher prevalence of diabetes among patients with severe COVID-19 as compared to patients with non-severe disease (16.2% *vs* 5.7%) (45). The China Center for Disease Control and Prevention reported a diabetes prevalence of 5.3% among all 44,672 COVID-19 cases, but 19.7% among non-survivors, with a case fatality rate of 2.3% *vs* 7.3%, respectively (27). In the USA, among 5,279 patients diagnosed with COVID-19 in a prospective cohort study in New York City, prevalence of diabetes was higher in patients admitted to the hospital than patients not admitted (34.7 *vs* 9.7%) (7). The US Centers for Disease Control and Prevention reported diabetes prevalence rising with increasing severity of COVID-19, from 6.4% in non-hospitalized patients to 24.2% in hospitalized patients and 32.4% in ICU patients (28). The most recent report of the Istituto Superiore di Sanità (National Health Institute) in Italy, describing 85,418 patients who died from COVID-19, reported a prevalence of T2D of 29.3% (46).

Apart from reporting a higher prevalence of diabetes, several meta-analyses showed that the risk of diabetes was 2 – 4 fold higher in severe COVID-19 cases as compared to non-severe COVID-19 patients (29, 43, 47). In a meta-analysis of six Chinese studies, the pooled rate ratio of diabetes among patients with severe versus non-severe COVID-19 was 2.26 (30). A retrospective cohort study on 178 patients with diabetes hospitalized with COVID-19 in the USA found that patients with diabetes had a 59% higher risk of ICU admission, an approximately 97% increased risk of mechanical ventilation and twofold increased risk of death after adjustment for age, sex, ethnicity, BMI and any comorbidities (48). In a final large meta-analysis of 76 studies involving 31,067 patients with COVID-19, patients with diabetes were at significantly higher risk of severe infection (OR 2.38) as well as mortality (OR 2.21) (49).

Since most research had either been done in patients with T2D or the researchers did not specify the type of diabetes in their studies, it had long been unclear whether this higher risk of a severe course of COVID-19 was also present in T1D. Fortunately, more evidence on the risk in T1D is emerging and it demonstrates an increased risk for COVID-19-related mortality, ICU admission and hospitalization. A population-based study from the United Kingdom (UK) demonstrated that out of 23,804 COVID-19-related in-hospital deaths, 31.4% of patients had T2D and 1.5% had T1D. Unadjusted 72-day mortality rates per 100,000 people were 27 for individuals without diabetes, 138 for T1D and 260 for T2D. Interestingly, adjusted for age, sex, socioeconomic deprivation, ethnicity and geographical region, the OR for COVID-19-related mortality was even higher in patients with T1D compared to patients with T2D (3.51 *vs* 2.03) (9). Even more recently, a similar nationwide population-based study on 34,383 patients with T1D and 275,960 with T2D from Scotland showed similar increased risks for COVID-19-related mortality and ICU admission, with again higher risks for T1D as compared to T2D (T1D OR 2.40; T2D OR 1.37) (50). A final recent prospective cohort study from the USA confirmed these findings (51), and showed in their latest analyses on an even higher number of patients that there was a higher risk of hospitalization for COVID-19 in T1D (OR 4.60) as compared to T2D (OR 3.42) (52). The findings of these studies are summarized in **Table 1**.

**Table 1:** Overview of the risk of adverse COVID-19-related outcomes in patients with type 1 and type 2 diabetes mellitus.

	Study population	Number of patients (n)	Outcome	T1D *vs* no DM OR (95% CI)	T2D *vs* no DM OR (95% CI)
Barron et al. (9)	United Kingdom, nationwide population-based	T1D: 263,830 T2D: 2,864,670	Mortality	3.51 (3.16 – 3.90)^#^ 2.86 (2.58 – 3.18)^$^	2.03 (1.97 – 2.09)^#^ 1.80 (1.75 – 1.86)^$^
	*March 1^st^ – May 11^th^ 2020*	No DM: 58,244,220			
McGurnaghan et al. (50)	Scotland, nationwide population-based	T1D: 34,383 T2D: 275,960	Mortality and/or ICU admission	2.40 (1.82 – 3.16)^&^	1.37 (1.28 – 1.47)^&^
	*March 1^st^ – July 31^st^ 2020*	No DM: 5,143,951			
Gregory et al. (51, 52)^Φ^	Nashville, Tennessee, USA, single-center*	T1D: 136 T2D: 1,100	Hospitalization	4.60 (3.04 – 6.98)^€^	3.42 (2.94 – 3.99)^€^
	*March 17^th^ – December 24^th^ 2020*	No DM: 19,422			

COVID-19, coronavirus disease 2019; T1D, type 1 diabetes; T2D, type 2 diabetes; DM, diabetes mellitus; OR, odds ratio; CI, confidence interval; ICU, intensive care unit; USA, United States of America.

^Φ^ Gregory et al. have published the initial findings of their prospective cohort study, followed by a report of further analyses on an even higher number of patients. Here, we report the findings of their latest analyses.

* Vanderbilt University Medical Center (VUMC), Nashville, Tennessee, USA.

^#^ Adjusted for age, sex, deprivation, ethnicity and geographical region.

^$^ Adjusted for age, sex, deprivation, ethnicity, geographical region and previous hospital admissions with coronary heart disease, cerebrovascular disease, or heart failure.

^&^ Adjusted for age and sex.

^€^ Adjusted for age, sex, ethnicity, hypertension, smoking, and body mass index (BMI).

In summary, patients with both type 1 and type 2 diabetes have an increased risk of a more severe course of COVID-19. While the risk for patients with T2D had already been established, recent evidence that emerged on the risk in T1D shows that patients with T1D appear to have an even slightly higher adjusted risk for adverse COVID-19-related outcomes. Although diabetes is an independent risk factor for disease severity (9, 48, 50–52), of course, patients with diabetes often have other comorbidities or concurrent factors that could add to their increased risk of severe COVID-19.

## Role of Cardiovascular Risk Factors, (Micro)vascular Complications and Pharmacologic Treatment

**Figure 1:**
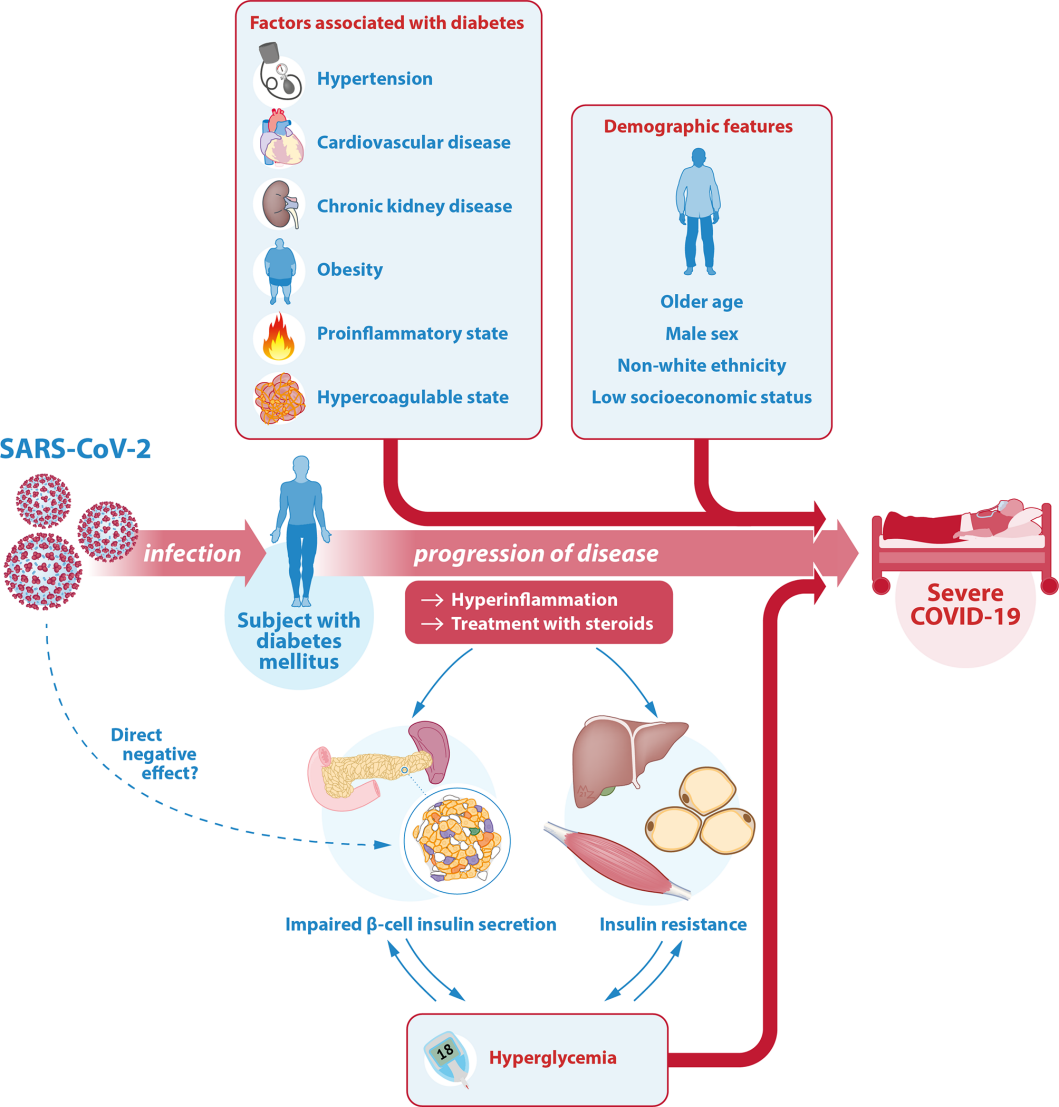
Illustration of the interrelationship between SARS-CoV-2, COVID-19 and diabetes. The relationship between COVID-19 and diabetes is complicated and bidirectional. Diabetes mellitus is one of the most important risk factors for severe COVID-19. In a patient with diabetes, the associated comorbidities and diabetes-related complications as well as certain demographic features can further contribute to this higher risk of a severe course of COVID-19. Another key factor is glycemic control. On the one hand, hyperglycemia is a strong risk factor for a more severe course of COVID-19. On the other hand, the hyperinflammation associated with severe COVID-19 as well as its treatment with steroids can cause or worsen hyperglycemia through an effect on insulin target tissues (predominantly liver, muscle and fat cells) reducing insulin sensitivity (insulin resistance), as well as on pancreatic β-cells causing insufficient insulin secretion. There may even be a direct effect of SARS-CoV-2 on the β-cell through the ACE-2 receptor, but this is controversial. Hyperglycemia itself can lead to glucose toxicity, thus further decreasing insulin sensitivity and insulin secretory function. Hereby, the risk of severe COVID-19 in patients with diabetes is increased even further. SARS-CoV-2, severe acute respiratory syndrome coronavirus 2; COVID-19, coronavirus disease 2019; ACE-2, angiotensin converting enzyme 2.

Several mechanisms have been suggested as an underlying additional explanation for the more severe course of COVID-19 in patients with diabetes. This chapter aims to provide an overview of prognostic factors associated with severity of COVID-19 in patients with diabetes. It focuses on the most relevant factors in the context of diabetes and COVID-19 such as demographic features, cardiovascular risk factors, (micro)vascular complications and pharmacologic treatments. Other comorbidities associated with diabetes mellitus are beyond the scope of this review (53). In assessing the risk of an individual for a more severe course of COVID-19, it is important to consider these prognostic features, summarized in the top half of **Figure 1**.

### Age, Sex, Ethnicity, Socioeconomic Status

In the general population, a higher age, male sex, non-white ethnicity and lower socioeconomic status are demographic features that have all been associated with a more severe course of COVID-19 (7, 8, 42, 47, 54–58). As expected, these features also add to the risk of COVID-19 severity in patients with diabetes. Similar to the non-diabetic population, in a Chinese two-center retrospective study on 306 patients hospitalized with COVID-19, older age and male sex were found more frequently in non-survivors as compared to survivors among patients with diabetes (age 72 *vs* 63 years; 71.0% *vs* 43.4% male, respectively). Age ≥ 70 years was found to be an independent risk factor for in-hospital death for patients with diabetes as well as for patients without diabetes (hazard ratio (HR) 2.39 and 5.87, respectively) (59). In a recent larger French nationwide prospective cohort study on 1,317 patients with diabetes hospitalized with COVID-19 (of which 88.5% T2D), age was an independent risk factor for 7-day mortality as well (OR 2.39) (60). In that study, patients with T1D (n = 56) were separately described, confirming that patients with T1D who met the primary outcome (i.e. requirement of mechanical ventilation or death by day 7) were significantly older compared to patients who did not (65.3 *vs* 53.2 years) and prevalence of this primary outcome incrementally increased with increasing age in patients with T1D (12.0% for age < 55 years, 23.8% for age 55 – 74 years and 50.0% for age ≥ 75 years) (61). The UK population-based study on a total of 264,390 patients with T1D and 2,847,020 patients with T2D confirmed increased COVID-19-related mortality in both T1D and T2D for all of the demographic features. As compared to patients 60 – 69 years of age, patients 70 – 79 years of age were at increased risk for death (T1D HR 1.89; T2D HR 1.94), and patients ≥ 80 years of age were at even greater risk (T1D HR 4.79; T2D HR 4.52). For male sex, risk of mortality increased similarly for patients with T1D as compared to T2D (HR 1.61 for both). In T1D, COVID-19-related mortality was significantly higher in individuals of black and Asian ethnicities compared to white ethnicity (HR 1.77 and 1.57, respectively). For patients with T2D, risk of COVID-19-related mortality was higher in individuals of black and Asian, as well as mixed ethnicity (HR 1.63, 1.08 and 1.30, respectively). The researchers also identified a clear association between COVID-19-related death and socioeconomic status among patients with either type of diabetes, with mortality significantly higher in individuals in the most deprived quintile as compared to those in the least deprived quintile (T1D HR 1.93; T2D HR 1.46) (10). The Scottish population-based study on 319,349 patients with diabetes found similar associations for age (OR 1.076), sex (female OR 0.705) and socioeconomic status, with risk of ICU admission or mortality for COVID-19 decreasing with each quintile of increasing socioeconomic status (least deprived quintile *vs* most deprived quintile OR 0.379) (50). Additionally, risk for hospitalization in patients with diabetes was also found to be increased in the USA prospective cohort study for age (OR 1.93 for each 25 year increment), sex (OR 1.22 for male) and ethnicity (OR 2.01 for black; OR 1.78 for other) (51).

### Hypertension and Cardiovascular Disease

Among the most commonly described comorbidities present in patients with COVID-19 are hypertension and cardiovascular disease (CVD), with hypertension being the most common in numerous reports from around the world (6–8, 24–28, 42, 43, 45–47, 54, 56). Hypertension and CVD are also among the most commonly associated comorbidities with diabetes (3, 4). Considering this strong association, and the fact that both hypertension and CVD have been identified as strong independent risk factors for severity of COVID-19 in the general population (47, 56, 58), it can be safely assumed that they are important concomitant factors adding to the risk of severe COVID-19 in patients with diabetes. Several studies have confirmed this. In a Chinese study on hospitalized COVID-19 patients, 153 individuals with diabetes more frequently had a history of hypertension and CVD as compared to 153 individuals without diabetes who were age- and sex-matched (hypertension 56.9% *vs* 28.8%; CVD 20.9 *vs* 11.1%, respectively). Among non-survivors, 83.9% of patients with diabetes suffered from hypertension and 45.2% from CVD, compared to 37.5% and 18.8% of patients without diabetes. In this cohort, hypertension was independently associated with an increased risk for in-hospital COVID-19-related death in patients with diabetes (HR 3.10) (59). A multi-center retrospective cohort study from Italy confirmed hypertension to be associated with an additional increased risk of mechanical ventilation, ICU admission or death (OR 2.31) in patients with diabetes (62), and a prospective cohort study from the USA found increased adjusted risk for hospitalization (OR 1.39) (51), although this was not described in every cohort (10, 50). In the Chinese study, as opposed to hypertension, CVD was only found to be significantly related to in-hospital death in patients with diabetes in the univariable regression analyses (HR 3.79), but not in the adjusted multivariable analyses. In the French nationwide cohort study on patients with COVID-19, an unadjusted increased risk for death was identified for patients with diabetes and concomitant hypertension (OR 1.81), macrovascular complications (OR 3.58), microvascular complications (OR 5.25), and several other cardiovascular-related diseases (60). Because T2D and CVD are so strongly related, it has been suggested that the higher risk of severe COVID-19 for T2D and CVD may overlap and not be additive. In the only currently available case-control study from Italy on 79 patients with T2D hospitalized for COVID-19 and 158 age- and sex-matched T2D controls without COVID-19, no higher prevalence of CVD was found in cases with COVID-19 (63). However, in a multi-center retrospective cohort study from the same group, a single cardiometabolic risk factor did not significantly increase risk of critical COVID-19 or mortality, but cardiometabolic multimorbidity did significantly and independently increase this risk (OR 3.19) (62). In the UK population-based study, for T1D, previous stroke (HR 2.33) and previous heart failure (1.80) but not previous myocardial infarction were associated with COVID-19-related mortality. For T2D, all of these cardiovascular comorbidities were independently associated with increased risk of death (myocardial infarction HR 1.11; stroke HR 2.02; heart failure HR 2.09) (10).

### Chronic Kidney Disease

Chronic kidney disease (CKD) is one of the well-known microvascular long-term complications of diabetes. Also, diabetes is the most common cause of chronic kidney disease (CKD). The prevalence of CKD among patients with T2D is around 40% (64, 65) and the lifetime risk of developing CKD in T1D around 20-25% (66). In patients hospitalized with COVID-19, CKD has been shown to be an independent risk factor for in-hospital death (58, 67). Recent studies have demonstrated that this association is also present in patients with diabetes. In the French nationwide cohort study on hospitalized COVID-19 patients, diabetic kidney disease (i.e. estimated glomerular filtration rate (eGFR) of ≤ 60 mL/min/1.73m^2^ and/or proteinuria) was a predictor for early death in patients with diabetes (OR 3.19) (60). Additionally, the Italian case-control study on patients with T2D hospitalized with COVID-19 and T2D controls without COVID-19 confirmed a higher prevalence of CKD stage IIIb (eGFR < 45 ml/min/1.73m^2^) in those hospitalized (OR 3.09) (63). In the UK population-based study, an eGFR < 60 mL/min/1.73m^2^ was associated with increased COVID-19-related mortality in both T1D and T2D. Increasing stages of CKD were associated with incrementally increasing risks of COVID-19-related mortality, for eGFR 45 – 59 mL/min/1.73m^2^ (T1D HR 2.07; T2D HR 1.39), eGFR 30 – 44 mL/min/1.73m^2^ (T1D HR 2.46; T2D HR 1.76), eGFR 15 – 29 mL/min/1.73m^2^ (T1D HR 3.71; T2D HR 2.31) and finally eGFR < 15 mL/min/1.73m^2^ (T1D HR 8.35; T2D HR 4.91) (10). It is important to recognize CKD in patients with COVID-19 because impaired renal function warrants dosage reduction of some treatments used for COVID-19 (68).

### Obesity

Obesity has been extensively linked to severity and mortality of COVID-19 (7, 58, 69–73). Several mechanisms have been suggested to explain the strong association between obesity and a severe course of COVID-19, i.e. ventilatory difficulty, the hypercoagulable and proinflammatory environment present in obesity, alterations in gut microbiota, nutritional deficits and immune dysregulation (74). Obesity is also linked to diabetes, in particular T2D, with an association so strong that the term diabesity has been used in the literature (75). Individuals with increasing body mass index (BMI) have an incrementally increasing risk of developing diabetes, already starting at an overweight BMI (25 – 29.9 kg/m^2^; OR 1.59), through class 2 obesity (BMI 30 – 39.9 kg/m^2^; OR 3.44) up to class 3 obesity (BMI ≥ 40 kg/m^2^; OR 7.37) (76). With both diabetes and obesity providing proinflammatory, hypercoagulable and immune dysregulatory conditions, an additional effect of obesity on severity of COVID-19 in patients with diabetes can be expected. Indeed, obesity has been shown to increase the risk of a severe course of COVID-19 in patients with diabetes. In the French nationwide cohort study, BMI was independently associated with their primary outcome (tracheal intubation and/or death within 7 days; OR 1.24). Interestingly, this effect was not significant in COVID-19-related death alone (60). The USA prospective cohort study found each 8.9 kg/m^2^ increment to be associated with an adjusted OR of 1.25 for hospitalization (51). An effect on COVID-19-related mortality was found in the UK population-based study, which identified a U-shaped association between BMI and mortality for both T1D and T2D. In that study, obesity was independently associated with an even higher additional risk of death in patients with T1D compared to T2D. Compared with a BMI of 25.0 – 29.9 kg/m^2^, a lower BMI of < 20.0 kg/m^2^ as well as of 20.0 – 24.9 kg/m^2^ were associated with increased COVID-19-related mortality (T1D HR 2.45 and 1.51; T2D HR 2.33 and 1.34, respectively). A higher BMI of 35.0 – 39.9 kg/m^2^ as well as of ≥ 40.0 kg/m^2^ also accounted for increased risk of COVID-19-related mortality for both types of diabetes (T1D HR 1.72 and 2.33; T2D HR 1.17 and 1.60, respectively). Interestingly, for T1D but not for T2D, a BMI of 30.0 – 34.9 kg/m^2^ was additionally associated with mortality (HR 1.47) (10). This U-shaped association with a low BMI, in addition to an high BMI, also attributing to an increased risk of death may be an explanation for the lack of significance on mortality in the French cohort in which a BMI of < 25 kg/m^2^ was set as the reference for statistical analyses.

### Inflammation

In COVID-19, the most common post-mortem findings include profound inflammation of several tissues (77). Patients with severe COVID-19, in particular patients with acute respiratory distress syndrome, have a marked rise in inflammatory markers such as C-reactive protein (CRP), D-dimer, ferritin and interleukin 6 (IL-6) (8). An extreme form of hyperinflammation associated with severe COVID-19 is found in some patients who experience a cytokine storm, an uncontrolled state of hyperinflammation resulting in widespread tissue damage, multi-organ failure and death (78, 79). In several studies, high blood concentrations of inflammatory markers, cytokines and chemokines have been associated with COVID-19 severity and mortality (7, 8, 42, 80, 81). Diabetes is associated with a proinflammatory state, which may contribute to the risk of a more severe course of COVID-19 and a higher risk of experiencing a cytokine storm. The proinflammatory cytokines and toxic metabolites that are present in a cytokine storm are already chronically elevated in individuals with diabetes as part of the low-grade chronic inflammation (82–84). Although understanding the underlying pathogenesis of low-grade inflammation leading to a more rapid progression of COVID-19 and the associated cytokine storm is still subject of research, one of the most important pathophysiological mechanisms underlying the higher risk in patients with diabetes is thought to involve the proinflammatory NF-kappa-B pathway, which is chronically activated in patients with diabetes (82, 85). Additionally, the low-grade chronic inflammation present in individuals with diabetes is associated with exaggerated macrophage, monocyte and T-cell recruitment, and with decreased regulatory T-cell function, which promotes further inflammation in a continuous feedback cycle (86–89). Indeed, in several clinical studies in patients with diabetes and COVID-19, worse inflammatory profiles with higher inflammatory markers such as CRP, D-dimer, IL-6 and ferritin were identified compared to patients without diabetes (17, 19, 44, 90). Patients with diabetes were also at a higher risk of excessive uncontrolled inflammation responses (90). Additionally, elevated CRP and D-dimer levels, as well as higher IL-6, TNF-α and ferritin levels were found in non-survivors as compared to survivors with diabetes (44, 59). A high CRP was independently associated with increased risk of mortality (OR 1.87) for patients with diabetes hospitalized with COVID-19 (60). Among patients with diabetes, the group most vulnerable to hyperinflammation and cytokine storm in COVID-19 consists of the individuals with poor glycemic control, since hyperglycemia stimulates the synthesis and release of cytokines (91).

### Coagulation

COVID-19 has been widely associated with thromboembolic events such as pulmonary embolism, deep-vein thrombosis (DVT), ischemic stroke and myocardial infarction, which represent a predominant cause of death in patients with severe COVID-19 (92–94). Similar to the proinflammatory state, diabetes is also associated with a hypercoagulable state. Patients with diabetes in general have an increased risk of thromboembolic events (95), which, in the case of COVID-19, can add to a high risk of death. Hypercoagulation in COVID-19 is thought to occur due to the profound inflammatory response and cytokine storm observed in some patients. Because patients with diabetes have a more pronounced inflammatory response (see paragraph *Inflammation*), they may be at greater risk to suffer from thromboembolic events in the case of COVID-19. Another important contributor in patients with diabetes may be hyperglycemia, which was previously shown to further exaggerate coagulation, as well as hyperinsulinemia, which inhibits fibrinolytic activity (96). In two studies, longer prothrombin times and higher D-dimer concentrations were found in COVID-19 non-survivors as compared to survivors in hospitalized Chinese patients with diabetes (44, 59). However, this was not the case in the French nationwide cohort study in which D-dimer levels were not a significant predictor of 7-day mortality in patients with diabetes hospitalized with COVID-19 (60). Apart from these studies describing biochemical outcome parameters such as D-dimer levels, no clinical studies have yet been performed on the prevalence of thromboembolic events in patients with diabetes as compared to patients without diabetes. It could be hypothesized that patients with COVID-19 and diabetes are at higher risk of thromboembolic events, especially when other risk factors such as older age, obesity, inflammation and immobilization due to hospital admission are present.

### Medication

In addition to diabetes mellitus and the conditions that are associated with the disease, the treatments of these conditions might also increase the risk of a more complicated course of COVID-19, as well as account for drug interactions with treatments given for COVID-19. This gives rise to some specific considerations in patients with diabetes and/or associated comorbidities who are infected with SARS-CoV-2. The benefits, risks and recommendations for each of the treatments are summarized in **Table 2**.

**Table 2:** Benefits, risks and recommendations for glucose-lowering and other commonly used treatments in patients with diabetes and COVID-19.

Medication	Benefits	Risks	Recommendations
***a. Glucose-lowering drugs***			
Insulin	Careful adjustment for improved glucose regulation possible, associated with better COVID-19 outcomes.	Hypoglycemia, especially in combination with (hydroxy)chloroquine. High dosages could be needed in critically ill patients, especially when treated with steroids.	Do not discontinue, initiate in patients with severe COVID-19.
Metformin	Anti-inflammatory properties, no hypoglycemia.	Lactic acidosis in critically ill patients.	Discontinue in patients with a severe course (impaired renal or hepatic function, sepsis, heart failure, respiratory distress).
Sulfonylureas	N/A	Hypoglycemia, especially in combination with (hydroxy)chloroquine.	Consider discontinuation based on COVID-19 severity, nutritional status and risk of hypoglycemia.
SGLT-2 inhibitors	No hypoglycemia.	Diabetic ketoacidosis and dehydration.	Discontinue in patients with a severe course (impaired renal or hepatic function, sepsis, heart failure, respiratory distress).
GLP-1 receptor agonists	Anti-inflammatory properties, no hypoglycemia.	Risk of dehydration with side-effects like vomiting and diarrhea.	Do not discontinue, encourage adequate fluid intake and regular meals.
DPP-4 inhibitors	Well-tolerated, no hypoglycemia, usable in wide range of renal function.	N/A	Do not discontinue.
Thiazolidinediones	Anti-inflammatory properties, reduction of insulin resistance.	Risk of fluid retention and aggravation of heart failure.	Consider discontinuation in patients with a severe course, particularly patients with heart failure.
***b. Other commonly used drugs***			
Statins	Possible beneficial effect on COVID-19-related outcomes, at least no detrimental effects.	N/A	Do not discontinue.
ACE-inhibitors/ARBs	Possible beneficial effect on COVID-19-related outcomes, at least no detrimental effects.	N/A	Do not discontinue.

N/A, not applicable; SGLT-2, sodium-glucose-co-transporter-2; GLP-1, glucagon-like peptide 1; DPP-4, dipeptidyl peptidase 4; ACE, angiontensin-converting enzyme; ARB, angiotensin receptor blocker.

Some studies reported that insulin use in patients with diabetes was associated with a greater COVID-19 related morbidity and mortality (50, 60, 97). Even though in two of these studies adjustment for confounders using multivariable regression models was performed (50, 60), worse outcomes in patients on insulin therapy could be related to confounding by indication, rather than pointing to a harmful effect of insulin (98). Patients on insulin therapy are more likely to have a more severe and a longer duration of diabetes. In addition, glucose dysregulation, and thus increased use of insulin, occurs with more severe COVID-19 (see paragraph *Direct and Indirect Effects of SARS-CoV-2*). Indeed, better glycemic control is associated with better COVID-19-related outcomes (see paragraph *Glycemic Control*). In one study, treatment with insulin was associated with achievement of glycemic targets and improvement of COVID-19-related outcomes in patients hospitalized with the disease (99). The French nationwide cohort study also did not find any significant association between insulin use and increased mortality (60). Moreover, the Italian case-control study on patients with T2D hospitalized with COVID-19 and T2D controls without COVID-19 even demonstrated a lower prevalence of basal insulin use in those hospitalized (OR 0.18), after adjustment for cardiovascular, pulmonary and kidney disease (63). Consensus recommendations of experts from around the world include the recommendation that insulin therapy should not be stopped, as well as the recommendation to start insulin therapy in patients with severe COVID-19 (100). Of course, in patients with T1D, cessation of insulin therapy is never an option. Steroids, an important treatment pillar for patients with severe COVID-19, can result in marked hyperglycemia and the need for very high dosages of insulin.

Patients with COVID-19 that were treated with metformin have been reported to have better outcomes including reduced mortality and lower levels of inflammation in studies using univariable (97), as well as adjusted multivariable analyses (60). This was consistent with previous findings of anti-inflammatory properties of metformin (101), although improved COVID-19-related outcomes were not found in every study (50). However, metformin poses a known risk of lactic acidosis in critically ill patients, including patients with COVID-19 (102). It has therefore been recommended to discontinue metformin in patients with a severe course of COVID-19, i.e. in patients with sepsis, impaired renal or hepatic function, heart failure or respiratory distress (100, 103). However, because of the anti-inflammatory, cell-protective and important glucose regulatory effects of metformin without risk of hypoglycemia, it has been suggested to maintain metformin treatment in all patients hospitalized with COVID-19, provided they have not developed kidney or liver failure (104).

With contradictory evidence, it is still unclear whether sulfonylureas are associated with increased morbidity or mortality in patients with COVID-19. In the Scottish nationwide population-based study, the authors have found an increased risk of severe COVID-19 in patients using sulfonylureas (OR 1.310) after adjustment for age, sex, diabetes duration and type of diabetes (50). In an unadjusted univariable analysis from the French nationwide cohort study, this effect was not found (60). As sulfonylureas convey an increased risk of hypoglycemia, which increases during illness if oral intake is poor, discontinuation of sulfonylureas may be warranted in certain patients with COVID-19, based on the severity of COVID-19, nutritional status and risk of hypoglycemia.

Sodium-glucose-co-transporter-2 (SGLT-2) inhibitors do not carry a risk of hypoglycemia and have not been associated with increased risk of severe COVID-19 in multivariable adjusted analyses (50). However, they are associated with a risk of diabetic ketoacidosis during illness (105, 106). To reduce the risk of metabolic decompensation, expert recommendations state to discontinue SGLT-2 inhibitors in patients with severe COVID-19 (100).

Glucagon-like peptide-1 (GLP-1) receptor agonists exert anti-inflammatory properties, which is known to have beneficial effect on low-grade inflammation associated with atherosclerosis (107, 108). Furthermore, GLP-1 receptor agonists have renoprotective effects (109, 110). Considering the well-known beneficial effect of both GLP-1 receptor agonists as well as SGLT-2 inhibitors on prevention of cardiovascular and kidney disease, both risk factors for a severe course of COVID-19, these drugs may be of preventive importance (111). Whether GLP-1 receptor agonists exert similar beneficial anti-inflammatory effects specifically in relation to inflammation in COVID-19 is not yet clear. In several animal studies, GLP-1 receptor agonists have been associated with attenuation of pulmonary inflammation and preservation of lung function in experimental lung injury as well as respiratory syncytial virus infection (112–115). In the French prospective study on patients with COVID-19, use of GLP-1 receptor agonists was, however, not associated with a change in mortality (60). In the Scottish nationwide population-based study, adjusted risk of severe COVID-19 was even found to be increased (50). GLP-1 receptor agonists do not pose a risk for hypoglycemia, but the gastro-intestinal side-effects of GLP-1 receptor agonists may cause or worsen dehydration. Adequate fluid intake and regular meals should be encouraged. Cessation of GLP-1 receptor agonists in patients with COVID-19 is not deemed necessary (100).

Dipeptidyl peptidase-4 (DPP-4) inhibitors have been subject to debate since the start of the COVID-19 pandemic. In previous coronaviruses, DPP-4 was found to be a co-receptor for viral entry into the cell (116). Therefore, it had been speculated that DPP-4 inhibitors might have a beneficial effect, interfering with this binding and therefore reducing viral entry (117, 118). One retrospective cohort study found reduced mortality and improved clinical outcomes in patients with T2D and COVID-19 using sitagliptin (119). However, multiple clinical studies did not show any morbidity or mortality benefit of DPP-4 inhibitors in univariable (60, 97, 120), adjusted multivariable (50), or propensity-score matched analyses (121). In line with these findings, a recent study on SARS-CoV-2 showed that it does not use DPP-4 as a co-receptor for viral entry (122). Because DPP-4 inhibitors are generally well-tolerated, have no risk of hypoglycemia and can be used for a wide range of kidney function, it is recommended that they are continued in patients with COVID-19 (100).

Thiazolidinediones exert anti-inflammatory and antioxidant effects and significantly reduce insulin resistance (123). Therefore, it has been speculated that thiazolidinediones could ameliorate prognosis of COVID-19 in patients with diabetes (124). However, this has not yet been confirmed by clinical studies (50). Important side-effects of thiazolidinediones include weight gain and fluid retention, associated with aggravation of heart failure (125). For this reason, until more clinical trials more accurately identify the risks and benefits associated with thiazolidinedione treatment in (severe) COVID-19, use of thiazolidinediones in patients with diabetes and COVID-19 remains controversial. Discontinuation may be warranted in patients with a severe course of COVID-19, especially in patients with heart failure.

In addition to glucose-lowering therapies, patients with diabetes often also use other comedications like statins. Statins are lipid-lowering drugs that have notable anti-inflammatory and cardiovascular protective properties. Because CVD is a comorbidity highly associated with worse outcomes of COVID-19, it can be hypothesized that use of statins might reduce this risk (126). However, statins increase the expression of angiotensin converting enzyme 2 (ACE-2), the main receptor that is used by SARS-CoV-2 to enter a cell (127). This raised concerns on statins potentially increasing the risk for COVID-19 severity. To this end, several studies have looked into the effect of statins on COVID-19 morbidity and mortality. A large Chinese retrospective study on 13,981 patients with COVID-19 showed that in-hospital statin use was associated with reduced 28-day mortality as compared to a propensity score matched non-statin group (5.2% *vs* 9.4%, respectively) (128). In a retrospective cohort of 717 patients with COVID-19 in Singapore, after propensity score matching, statin use was independently associated with lower ICU admission, but not with a decrease in mortality (129). In the French prospective study on 1,317 hospitalized patients with diabetes and COVID-19, statins were also not associated with a change in mortality in the unadjusted analyses (60). In adjusted multivariable analyses of the UK population-based study, statins were not significantly associated with change in COVID-19-related mortality among patients with T1D. In T2D, use of statins was associated with a decrease in mortality (HR 0.72) (10). Although based on the available evidence it is still unclear whether statins have a beneficial effect on COVID-19-related outcomes, there at least does not seem to be a detrimental effect. Therefore, statins do not need to be discontinued in patients with COVID-19.

Because of their effect on the upregulation of the ACE-2 receptor (130), similar concerns as with statins were initially raised for ACE-inhibitors and angiotensin receptor blockers (ARBs) (131). Several studies have since then shown that there is no detrimental effect of ACE-inhibitors and ARBs on the severity of outcomes in patients with COVID-19 (60, 132–136). Some of the studies even showed improved clinical outcomes (50, 133, 136), although these findings need to be replicated in larger randomized controlled settings in order to draw a more definitive conclusion on any potential beneficial effects. In addition, with regard to a potential higher primary risk of infection, in a retrospective study on 4,480 patients as well as in a separate study on 12,594 patients who were tested for COVID-19, both using propensity score matching, the use of ACE-inhibitors and ARBs was not associated with a higher incidence of COVID-19 (135, 137). These findings are supported by a position statement of the European Society of Cardiology, that strongly recommends continuation of treatment with ACE-inhibitors and ARBs in patients with COVID-19 (138).

## Bidirectional Relationship: The Role of Glycemic Control and SARS-CoV-2

The interrelationship between COVID-19 and diabetes is complex. Apart from diabetes mellitus being one of the most important risk factors for severe COVID-19, with several comorbidities and diabetes-related complications further adding to this risk, another key factor is glycemic control. On the one hand, poor glycemic control is an important independent risk factor for a severe course of COVID-19. On the other hand, a severe SARS-CoV-2 infection can contribute to hyperglycemia through several mechanisms, thereby further worsening the prognosis of patients with COVID-19. This chapter is aimed to provide an overview of this bidirectional relationship, which is illustrated in the bottom half of **Figure 1**.

### Glycemic Control

Glycemic control as a predictor for worse outcomes of infections is not a new concept. During the 2009 H1N1 influenza pandemic, fasting plasma glucose levels were identified as an independent predictor for severity of H1N1 pneumonia (139). In the 2002 SARS-CoV outbreak, not only a history of diabetes (OR 3.0), but also fasting plasma glucose levels of ≥ 7.0 mmol/l (126 mg/dL) were found to be an independent predictor of mortality (OR 3.3) (39). Previous studies on the role of glycemic control in other infections have shown a U-shaped relationship between glycemia and infection-related hospitalization in 22,846 patients with T2D, with both low and high HbA1c levels increasing the risk of hospitalization, also after adjustment for confounding factors (14). In a population of 1,713 critically ill patients admitted to an intensive cardiac care unit, plasma glucose levels at admission were linearly associated with an increased risk for all-cause mortality (140).

In COVID-19, glycemic control also plays an important role in severity of outcomes. There are several different aspects to glycemic control that should be considered, i.e. glycemic control before hospital admission, at the time of hospitalization and during the in-hospital stay. An overview of important characteristics and outcomes of studies investigating the risk of adverse COVID-19-related outcomes according to glycemic control is presented in **Table 3**.

**Table 3:** Overview of the risk of adverse COVID-19-related outcomes according to glycemic control.

	Study population	Number of patients (n)	Parameter of glycemic control	Outcome	Risk HR/OR (95% CI)
***a. Glycemic control before hospitalization***					
Holman et al. (10)	United Kingdom, nationwide population-based cohort	T1D: 265,090 T2D: 2,889,210	HbA1c 59 – 74 mmol/mol (7.6 – 8.9%)	Mortality	T1D: HR 1.16 (0.81 – 1.67)* T2D: HR 1.22 (1.15 – 1.30)*
	*March 1^st^ – May 11^th^ 2020*		HbA1c 75 – 85 mmol/mol (9.0 – 9.9%)		T1D: HR 1.37 (0.90 – 2.07)* T2D: HR 1.36 (1.24 – 1.50)*
			HbA1c ≥ 86 mmol/mol (10.0%)		T1D: HR 2.23 (1.50 – 3.30)* T2D: HR 1.61 (1.47 – 1.77)*
Williamson et al. (58)	United Kingdom, nationwide population-based cohort	17,278,392	HbA1c ≥ 58 mmol/mol (7.5%)	Mortality	HR 2.61 (2.46 – 2.77)^#^ HR 1.95 (1.83 – 2.08)^$^
	*January 1^st^ – May 6^th^ 2020*				
Cariou et al. (60)	France, multi-center cohort *March 10^th^ – March 31^st^ 2020*	846	HbA1c 53 – 63 mmol/mol (7.0 – 7.9%) HbA1c 64 – 74 mmol/mol (8.0 – 8.9%) HbA1c ≥ 75 mmol/mol (9.0%)	Mortality	OR 1.55 (0.82 – 2.93)^&^ OR 1.09 (0.52 – 2.28)^&^ OR 0.84 (0.40 – 1.75)^&^
Gregory et al. (51, 52)^Φ^	USA, single-center cohort *March 17^th^ – December 24^th^ 2020*	T1D: 102	1^st^ HbA1c quartile 2^nd^ HbA1c quartile 3^rd^ HbA1c quartile 4^th^ HbA1c quartile	Hospitalization	OR 2.96 (1.11 – 7.86)^€^ OR 2.96 (1.11 – 7.86)^€^ OR 5.12 (2.12 – 12.35)^€^ OR 9.76 (4.42 – 21.54)^€^
***b. Glycemic control at the time of hospitalization***					
Wang et al. (141)	China, multi-center retrospective *January 24^th^ – February 10^th^ 2020*	605	Fasting blood glucose level ≥ 7.0 mmol/l (126 mg/dL)	Mortality In-hospital complications	HR 2.30 (1.49 – 3.55)^~^ OR 3.99 (2.71 – 5.88)^&^
Wu et al. (18)	China, multi-center retrospective *December 26^th^ 2019 – March 15^th^ 2020*	2,041	Hyperglycemia ≥ 6.1 mmol/l (110 mg/dL)	Critical disease and mortality overall Mortality in critical patients	HR 1.30 (1.03 – 1.63)^£^ HR 1.84 (1.14 – 2.98)^£^
Copelli et al. (17)	Italy, single-center retrospective	271	Hyperglycemia ≥ 7.78 mmol/l (140 mg/dL)	Critical disease and mortality	HR 1.80 (1.03 – 3.15)^¥^
	*March 20^th^ – April 30^th^ 2020*				
***c. Glycemic control during in-hospital stay***					
Bode et al. (20)	USA, multi-center retrospective	1,122	Diabetes and/or uncontrolled hyperglycemia (≥ 2 measurements > 10.0 mmol/l (180 mg/dL) within 24h)	Mortality	OR 6.12 (3.63 – 10.31)^&φ^
	*March 1^st^ – April 6^th^ 2020*				
Zhu et al. (19)	China, multi-center retrospective *December 30^th^ 2019 – March 20^th^ 2020*	Total: 7,337	Normoglycemia (glycemic variability during hospital stay 3.9 – 10.0 mmol/l (70 – 180 mg/dL) versus hyperglycemia (> 10.0 mmol/l (180 mg/dL)	Mortality	HR 0.14 (0.03 – 0.60)^ω^
		T2D: 952			
	

COVID-19, coronavirus disease 2019; T1D, type 1 diabetes; T2D, type 2 diabetes; HR, hazard ratio; OR, odds ratio; CI, confidence interval; USA, United States of America.

^Φ^ Gregory et al. have published the initial findings of their prospective cohort study, followed by a report of further analyses on an even higher number of patients. Here, we report the findings of their latest analyses.

* Adjusted for age, sex, socioeconomic deprivation, ethnicity, region of residence, duration of diabetes, body mass index (BMI), systolic blood pressure, prescription for antihypertensive drugs, serum total cholesterol, prescription for statins, smoking status, history of myocardial infarction, stroke, heart failure and eGFR.

^#^ Adjusted for age and sex.

^$^ Adjusted for age, sex, obesity, smoking status, deprivation, cancer, reduced kidney function, asthma, chronic respiratory disease, chronic cardiac disease, hypertension, chronic liver disease, stroke, dementia, other neurological disease, organ transplant, asplenia, rheumatoid arthritis, lupus or psoriasis and any other immunosuppressive condition.

^&^ Unadjusted.

^€^ Adjusted for age, sex, ethnicity, hypertension, smoking, and body mass index (BMI).

^~^ Adjusted for age, sex and CRB-65 score (measure of pneumonia severity).

^£^ Adjusted for age, sex, hypertension, smoking, insulin treatment, glucocorticoids, chronic kidney disease, chronic obstructive pulmonary disease, cancer and admission white cell counts, lymphocyte counts, D-dimer, aspartate transaminase, alanine transaminase and creatinine.

^¥^ Adjusted for age, sex, hypertension, cerebrovascular disease, chronic obstructive pulmonary disease, chronic kidney disease and cognitive impairment.

^φ^ Calculated from data provided in the original paper.

^ω^ Adjusted by propensity score matching, including age, sex, severity of COVID-19, hypertension, cardiovascular disease, cerebrovascular disease, chronic liver disease and chronic kidney injury.

#### Glycemic Control Before Hospital Admission

Patients with a pre-admission HbA1c of ≥ 86 mmol/mol (10.0%) compared to patients with an HbA1c of 48 – 53 mmol/mol (6.5 – 7.0%) had increased COVID-19-related mortality in both T1D and T2D (HR 2.23 and 1.61, respectively) (10). Interestingly, for T2D, a pre-admission HbA1c of 59 – 74 mmol/mol (7.6 – 8.9%) and of 75 – 85 mmol/mol (9.0 – 9.9%) were additionally associated with higher COVID-19-related mortality as compared to an HbA1c of 48 – 53 mmol/mol (HR 1.22 and 1.36, respectively) (10). A separate study from the UK on 17,278,392 individuals with 10,926 COVID-19-related deaths confirmed this finding, identifying HbA1c < 58 mmol/mol (7.5%) to yield a lower risk of COVID-19-related death as compared to HbA1c ≥ 58 mmol/mol (HR 1.58 *vs* 2.61, respectively, adjusted for age and sex) (58). Contrarily, in a French a nationwide cohort study on 1,317 patients with diabetes hospitalized for COVID-19, HbA1c was not associated with the composite outcome of mechanical ventilation and death (60). Additional analyses from the recent USA prospective cohort study showed in 102 patients with T1D that risk of hospitalization for COVID-19 incrementally increased with increasing levels of HbA1c (OR 2.96 for the first and second quartile of HbA1c, OR 5.12 for the third quartile and OR 9.76 for the fourth quartile) (51, 52).

During the pandemic, countries around the world have responded with different lockdown strategies in an attempt to control the outbreak. With glycemic control before hospital admission as an important risk factor for a severe course of COVID-19, several studies have been conducted on the effect of the pandemic and subsequent lockdown on glycemic control. It had been hypothesized that the limited possibility for physical exercise, increased stress and disruption of daily routines would negatively affect glycemic control. To this end, a large group of 280 patients with T1D and 155 patients with T2D was studied in our center during the lockdown period. Interestingly, we found an improvement in glycemic control in the lockdown period as compared to before the lockdown. In both T1D and T2D, patients in the highest pre-lockdown tertile of HbA1c showed the biggest improvement in HbA1c (T1D -4.3 mmol/mol (-0.39%); T2D -6.8 mmol/mol (-0.62%)). Improved continuous glucose monitoring data in patients with T1D reflected this observation (142). Smaller studies during the lockdown in Italy as well as Spain additionally supported this finding, with improved glucose time in range (3.9 – 10.0 mmol/l; 70 – 180 mg/dL), reduced time above range (> 10.0 mmol/l (180 mg/dL)) and reduced time below range (< 3.9 mmol/l (70 mg/dL)) in patients with diabetes who stayed at home during the lockdown (143–145).

#### Glycemic Control at the Time of Hospitalization

Hyperglycemia at the time of admission to the hospital was also identified as an important risk factor for a more severe course of COVID-19. Even in patients without previous diagnosis of diabetes, a high fasting blood glucose level ≥ 7.0 mmol/l (126 mg/dL) at the time of admission with COVID-19 was independently associated with 28-day mortality (141). A retrospective multi-center study of 2,041 COVID-19 patients hospitalized in China found hyperglycemia ≥ 6.1 mmol/l (110 mg/dL) at the time of hospital admission to be an independent risk factor for progression to critical disease and death in patients with non-critical illness at that time (HR 1.30). In patients diagnosed with critical disease, hyperglycemia ≥ 6.1 mmol/l (110 mg/dL) was an independent risk factor for in-hospital mortality (HR 1.84) (18). A study from Italy on 271 patients hospitalized with COVID-19 shows a stepwise increase in unadjusted mortality rates from patients without diabetes or hyperglycemia through patients with known diabetes and finally patients with at-admission hyperglycemia (16.8%, 28.6% and 39.4%, respectively), although the difference between patients with diabetes and hyperglycemia bordered on statistical significance. After adjustments, only hyperglycemia at the time of admission remained as an independent predictor of mortality (HR 1.80) (17).

#### Glycemic Control During In-Hospital Stay

Additionally, glucose regulation during in-hospital stay is another important prognostic factor for worse outcomes in patients hospitalized with COVID-19. In 1,122 patients hospitalized with COVID-19 in the USA, mortality rates were significantly higher in those with diabetes or uncontrolled hyperglycemia (i.e. ≥ 2 blood glucose measurements > 10.0 mmol/l (180 mg/dL) within a 24-hour period) during hospital stay as compared to those with normoglycemia (28.8% *vs* 6.2%, respectively) (20). In a Chinese retrospective multi-center study of 7,337 COVID-19 cases, of which 952 had T2D, patients with well-controlled blood glucose levels during hospitalization (glucose variability between 3.9 – 10.0 mmol/L (70 – 180 mg/dL)) were compared to patients with poorly controlled blood glucose levels (> 10.0 mmol/L (180 mg/dL)). Patients with good glycemic control during hospitalization had markedly lower mortality compared to poorly controlled patients, with an adjusted HR for mortality of 0.14 (19).

The strong influence of glycemic control and hyperglycemia on the severity of COVID-19 at all three different timepoints before as well as at the time of and during hospitalization, stresses the importance of glycemic control as a potential modifiable risk factor in patients with diabetes.

### Direct and Indirect Effects of SARS-CoV-2

Several clinical studies have shown that patients with COVID-19 are prone to notable glucose dysregulation. In patients with COVID-19, an unusually high number of diabetic ketoacidosis and hyperglycemic hyperosmolar syndrome have been reported (60, 146–150). Interestingly, new-onset diabetes has also been reported in patients hospitalized with COVID-19 (151–154). In one study on eight patients with T2D that were critically ill with COVID-19 and admitted to the ICU, insulin requirements rapidly increased to extremely high doses with a mean peak insulin requirement of 201 units per day (2.2 units/kg/day) (155).

This glucose dysregulation found in patients infected with SARS-CoV-2 may be the result of several mechanisms. First, the hyperinflammation and cytokine storm present in certain patients with a severe course of COVID-19 can have a substantial effect on insulin resistance (156). In addition, high dose steroids are recommended in patients with severe or critical COVID-19 by the WHO, as it considerably reduces the risk of mortality (157). Treatment with steroids is also well-known to increase peripheral insulin resistance, thereby inducing hyperglycemia (158). Another phenomenon that could play a role is stress hyperglycemia. In acute illnesses, cortisol, epinephrine and glucagon are released as a stress response, stimulating gluconeogenesis in the liver and thereby causing transient hyperglycemia (159, 160). The hyperglycemia resulting from these processes is likely to lead to glucose toxicity of β-cells, thereby further decreasing insulin secretory function. High dose steroids as well as inflammatory cytokines can additionally impair insulin secretion (161–163). These mechanisms may individually or collectively cause or worsen glucose dysregulation in patients with COVID-19.

Apart from these indirect mechanisms, it has been suggested that SARS-CoV-2 might have a direct effect on the pancreatic β-cells through interaction with the ACE-2 receptor. In order to enter a cell, SARS-CoV-2 binds with its spike protein to the ACE-2 receptor that is present on human epithelial cells of several tissues, including the small intestine and pulmonary alveoli (both transmission routes for SARS-CoV-2). After binding to the ACE-2 receptor, the virus’ spike protein is proteolytically cleaved by transmembrane serine protease 2 (TMPRSS2), which facilitates viral entry into the cells, where viral replication takes place leading to progression of the infection and cell-to-cell transmission (122). Previous research on the presence of the ACE-2 receptor in pancreatic β-cells and its role in coronavirus infection has been contradicting. Several studies have been performed after the 2002 SARS-CoV outbreak, since this virus, like SARS-CoV-2, also binds to the ACE-2 receptor. In a Chinese study, immunohistochemical staining of ACE-2 was performed on donated tissues of a 43-year old male brain-dead organ donor, including the pancreas. They showed clear staining of ACE-2 protein in the endocrine pancreatic islets of Langerhans, and nearly no staining in exocrine tissues (16). Another study used quantitative real-time polymerase chain reaction to quantitively map the transcriptional expression profile of ACE-2, and thereby found ACE-2 expression in the islets of Langerhans (164). However, neither of these studies have differentiated between the different cells present within the islets of Langerhans, i.e. the various endocrine cells as well as endothelial and ductal cells. In a recent study, immunohistochemistry showed ACE-2 expression particularly in the microvasculature, but also in ductal cells and in endocrine cells, where it was found to be preferentially expressed in β-cells (165). Conversely, a different study demonstrated ACE-2 expression in pancreatic microvascular and ductal cells, and only rarely in endocrine cells. They supported their finding by examining pancreata from three patients who died from COVID-19, where they found SARS-CoV-2 nucleocapsid protein in ductal epithelium, but not in endocrine cells (166). In an autopsy study on patients who died from SARS in the previous outbreak, SARS-CoV antigen and RNA were also not found in pancreatic endocrine cells (167). However, a recent study found that human pancreatic exocrine, but also endocrine cells express the viral entry receptor ACE-2, with highest staining coefficients in the pancreatic β-cells. The authors additionally showed that isolated human pancreatic islets, including β-cells, that were exposed to SARS-CoV-2 became infected and showed increasing intra- and extracellular viral RNA levels, pointing to SARS-CoV-2 viral replication. This was inhibited by co-culturing with remdesivir. In pancreatic β-cells that were infected, glucose-dependent insulin secretion was found to be impaired. These findings were supported by postmortem examination of different organs, including the pancreata, of four patients who died from COVID-19, staining for SARS-CoV-2 N-protein. This pancreatic histopathology revealed the presence of N-protein in both exocrine and endocrine cells in all four patients, indicating a persistent pancreatic SARS-CoV-2 infection during severe COVID-19 (168).

In summary, hyperglycemia is found in high numbers of patients with COVID-19, with mechanisms such as hyperinflammation, corticosteroid treatment, and glucose toxicity potentially contributing to this finding. Although current *in vitro* and *ex vivo* studies point to the possibility of SARS-CoV-2 infecting pancreatic β-cells, thereby impairing insulin secretion and having a direct effect on glucose dysregulation, the clinical relevance of these findings has not been established.

## Discussion

In summary, patients that suffer from diabetes mellitus do not appear to have an increased primary risk of COVID-19 infection, although several factors such as differences in social distancing behavior or willingness for SARS-CoV-2 testing make a robust conclusion difficult. Patients with both type 1 and type 2 diabetes are at greater risk for a severe course of COVID-19 and mortality. This poorer prognosis is likely additionally linked to the comorbidities and other risk factors that are often concomitantly present with diabetes mellitus, but also to glycemic control. The interrelation between diabetes and COVID-19 is complicated and bidirectional, with COVID-19 causing hyperglycemia on the one hand, but hyperglycemia causing worse outcome of COVID-19 on the other hand. Diabetes itself, as well as the comorbidities often associated with diabetes additionally contribute to this risk of a severe outcome of COVID-19. This relationship is illustrated in **Figure 1**.

Although diabetes itself appears to be an independent risk factor for severe COVID-19, the most important factors that co-contribute to an increased risk of COVID-19 severity and mortality in patients with diabetes include older age, hypertension, CVD, CKD, obesity, a proinflammatory and hypercoagulable state, and glucose dysregulation. All of these factors should be acknowledged when assessing the risk of a more severe course of COVID-19 in patients with diabetes. Patients with T1D appear to have a relatively higher risk of severe COVID-19 compared to T2D. Interestingly, the risk profile for patients with T1D appears to be different compared to the risk profile of those with T2D as well, at least for glycemic control and BMI, with an HbA1c ≥ 86 mmol/mol (10.0%) in T1D but ≥ 59 mmol/mol (7.5%) in T2D associated with increased COVID-19-related mortality. In both T1D and T2D, a BMI of < 25 kg/m^2^ increases the risk for death. However, in T1D a BMI of ≥ 30 kg/m^2^ is already associated with increased mortality, while in T2D this risk increases at a higher BMI of ≥ 35 kg/m^2^.

It is important to recognize that some of these risk factors are modifiable. For example, improved glucose regulation (i.e. better diabetes (self-)management) and a healthier BMI immediately contribute to a decreased risk of a severe course of COVID-19. It is imperative for health care professionals in the field as well as for patients with diabetes to be aware of the influence they herewith have on reducing their risk of COVID-19 severity as much as possible.

Although more knowledge is gradually surfacing as the pandemic progresses, challenges in understanding this interrelation between diabetes and COVID-19 remain. In particular, the clinical relevance of the potential direct effect of the virus on the function of pancreatic β-cells through the ACE-2 receptor, demonstrated in *in vitro* and *ex vivo* studies, requires further research. In addition, most research up until this moment has been performed in patients with T2D or in patients in whom the type of diabetes was not specified. The risk for T2D had therefore already been established. However, recently emerged evidence points to patients with T1D being at an even slightly higher risk of a more severe course of COVID-19 and increased mortality. Because of their completely different pathophysiology and the different comorbidities associated with either type of diabetes, future studies should distinguish between T1D and T2D to further understand specific risks.

## Author Contributions

EK initiated this study. CL designed the review structure, performed the literature search, selection and interpretation of data, and drafted the manuscript including tables and figures. CL and EK finalized and critically reviewed the manuscript and approved the final version. All authors contributed to the article and approved the submitted version.

## Conflict of Interest

The authors declare that the research was conducted in the absence of any commercial or financial relationships that could be construed as a potential conflict of interest.

## Abbreviations

ACE(-2), Angiotensin converting enzyme (2); ARB, Angiotensin receptor blocker; BMI, Body mass index; CKD, Chronic kidney disease; COVID-19, Coronavirus disease 2019; CRP, C-reactive protein; CVD, Cardiovascular disease; DPP-4, Dipeptidyl peptidase 4; eGFR, Estimated glomerular filtration rate; GLP-1, Glucagon-like peptide 1; HbA1c, Glycated hemoglobin A1c; HR, Hazard ratio; ICU, Intensive care unit; IL, Interleukin; MERS-CoV, Middle-East respiratory syndrome coronavirus; OR, Odds ratio; SARS-CoV(-2), Severe acute respiratory syndrome coronavirus (2); SGLT-2, Sodium-glucose-co-transporter-2; T1D, Type 1 diabetes mellitus; T2D, Type 2 diabetes mellitus; WHO, World Health Organization.
